# A qualitative assessment of the influence of family dynamics on adolescents’ sexual risk behaviour in a migration-affected community

**DOI:** 10.1080/17482631.2020.1717322

**Published:** 2020-01-24

**Authors:** Felix Chima Anyanwu, Henry Abayomi Akinsola, Augustine Kwame Tugli, Nkechi Obisie-Nmehielle

**Affiliations:** a Department of Public Health, School of Health Sciences, University of Venda, Thohoyandou, South Africa; b Department of Health Metrics, Applied Research for Community Development (ARCD), Limpopo, South Africa; c Department of Nursing Science, Lead City University, Ibadan, Nigeria; d Department of Migration Health, International Organization for Migration (IOM), Pretoria, South Africa

**Keywords:** The family, family dynamics, adolescents, sexual risk behaviour, migration-affected communities

## Abstract

**Purpose:** Adolescents may be known to take risks, but they may also conform to societal norms if they are given proper guidance, implying that there is a need for proper monitoring. This qualitative study explores the influence of family dynamics on adolescents sexual risk behaviour within a migration affected community.**Method:** Using thematic analysis, we processed data collected from 13 adolescents and 10 parents.**Results:** The themes generated from our data include the following, “Household poverty”, “Family conflicts”, “Lack of discipline”, “Parent-child closeness”, “Generational gap between adolescents and their parents” and “Lack of sex education”. Our study found that financial constrain was a major problem in this community, the impact of this was worse felt among adolescents who lived by themselves. In addition, some parents were delinquent, distant or detached from their adolescents. We also found evidence to suggest that couple conflict was a common occurrence, and this had negative influence on adolescent well-being and there sexual behaviour.**Conclusion:** In view of the deepening crisis of early sexual engagement among adolescents and the accompanying risk of unplanned pregnancy and sexually transmitted infections, it is imperative to foster adolescent friendly households where the parents/caregivers are empowered to support their adolescents.

## Introduction

Adolescence is often associated with sexual risk behaviour, and this association remains significant irrespective of the geographical placement or ethnic background of the adolescent (Malhotra, 2008). However, the proportion of adolescents engaging in sexual risk behaviour may differ across societies. The reason for these differences may also differ depending on the acceptable societal norms, culture and traditions (Malhotra, 2008). Although sexual risk behaviour is common among adolescents (Malhotra, 2008; WHO, 2014a), the adjourning circumstances and experiences faced by each generation of adolescents are different from that of the generation before them (WHO, 2014b). Furthermore, the untoward outcomes of sexual risk taking have been shown to have far-reaching implications, and this is because engaging in early sexual intercourse has been associated with increased rates of Sexually Transmitted Infections (STIs) and teenage pregnancy (Malhotra, 2008; Michigan Department of Education, 2007). It has also been shown that early sexual intercourse increases the likelihood of engaging in other sexual risk behaviours (IOM & NRC, 2011; Sandfort, Orr, Hirsch, & Santelli, 2008) such as inconsistent use of condom, which is an important determinant for STIs, including HIV infection (Schantz, 2012).

Empirical evidence has shown that risk behaviour and poor decision-making during adolescence are associated with biological, cognitive and social transitions experienced by young people as they grow into adulthood (AACAP, 2011; de Guzman & Pohlmeier, 2014; WHO, 2014a). Although these transitions may provide plausible explanations for the risk behaviour in adolescence, there are pockets of argument that suggest that adolescents face a greater challenge from other factors that are beyond their control. This assertion stems from varied studies that have examined the factors that influence adolescents’ behaviour (Alimoradi, Kariman, Simbar, & Ahmadi, 2017; Malhotra, 2008; NardI, Cunha, Bizarro, & Dell’aglio, 2012).

According to Clark (2002), adolescents live in a constantly changing world, a world wherein the socio-economic and cultural environment is ever-changing, differing from one society to another, and this may contribute to the often reported untoward behaviour of adolescents who indeed may be struggling to adjust and respond to these intra-personal and extra-personal changes. Indeed, attempts by adolescents to respond to this evolving socio-cultural environment may result in lifestyle changes that divert from the desired health trajectory for their optimal growth and development (Clark, 2002).

However, it is important to note that although adolescents are known to take risks, but they may also conform to societal norms if they are given proper guidance (Estrada-Martınez, Padilla, Caldwell, & Jo Schulz, 2011), showing that there is a need for adequate and proper monitoring and supervision by adults within the family and the society at large. In the light of this, the family as a social unit is primarily charged with the responsibility to provide the needed guidance for these individuals. However, the traditional family structure is buckling under the effects of globalization, modernization and industrialization, resulting in changes in family structure and composition. In some instances, the family is separated along geographical divides due to the migration of breadwinners to more economically advanced cities in search of better-paying jobs (Safta, Stan, Iurea, & Suditu, 2014). The resulting family separation, its family de-structuring consequences and the reversal of gender role may result in lack of proper monitoring, guidance and supervision for the adolescent (Safta et al., 2014). For instance, it has been suggested that the family structure impacts on the parenting processes (Gayles, Coatsworth, Pantin, & Szapocznik, 2009). As shown by Gayles et al. (2009), a single-parent-led family may be constrained with taking decisions and enforcing rules in order to discipline or support a child’s behaviour compared to dyadic families.

Notwithstanding the structural composition of the family, the style of parenting has played a huge role in shaping adolescent behaviour. For instance, parenting style has been part of discussing dominating policy reforms targeted at achieving better health outcomes for adolescents. Literature has shown that compared to authoritarian and permissive parenting styles, the authoritative parenting style is the most protective with regards to adolescent risk behaviour (Becona et al., 2012; Domenech Rodriłguez, Donovick, & Crowley, 2009). This is because parents who practice authoritative parenting are more likely to understand their adolescents, respond promptly to their needs, demand more accountability and allow more autonomy to the adolescents. These qualities promote mutual respect and creates an opportunity for effective communication between parents and their adolescents. According to Oswalt (2015), parent–child closeness is an important factor in fostering healthy adolescent development. However, it has been shown that the closeness between parents and their children begins to wane as the children begin to attain adulthood (Oswalt, 2015). Therefore, parents who remain distant from their children may create an opportunity for adolescents to bond strongly with their peers who may influence them negatively (O’Donnell et al., 2008).

Family dynamics are important determinants that define adolescents’ health trajectories, these family factors are particularly harsh in communities where families struggle with conflicts, separation and unemployment. Families in migration-affected communities like the mining towns in South Africa are under pressure to meet their obligation to their children due to their low socioeconomic status (Cronjé, Reyneke, & Van Wyk, 2013). This has prompted the implementation of public health interventions to mitigate the impact of negative family environment on adolescent development. Some of these public health programmes have targeted adolescents, while some have focused on strengthening the family unit in order to support the adolescent (Elkington, Bauermeister., & Zimmerman., 2011; Kirby, Coyle, Alton, Rolleri, & Robin, 2011). However, there have been many inconsistencies with regards to the impact of these programmes in the receiving communities (Chin et al., 2012). Perhaps, the reason for these inconsistencies may arise from the design and implementation of these programmes, some of which are developed and evaluated in communities that are non-homogenic to the receiving community (Goesling, Colman, Trenholm, Terzian, & Moore, 2013). Therefore, it is important to design and implement evidence-based programmes targeted at the factors that have a direct impact on adolescent sexual risk behaviour in a specified population and shy away from a one-fit-all approach (Goesling et al., 2013; Kirby et al., 2011).

This paper explores the influence of family dynamics on adolescents sexual risk behaviour within a migration-affected community in South Africa.

## Methodology

This study was approached from a purely qualitative perspective using exploratory design, taking into account the aim of the study which focused on the intricate issues around family dynamics and its impact on the adolescent. This design was used to inquire how family members within the community relate with one another and how the level and quality of relationship and support within the family rub off on the adolescents.

## Study setting

This study was conducted in Thabazimbi, a mining community in the Waterberg District in the Limpopo Province, South Africa. This community is richly populated by indigenous and migrant families from across South Africa and beyond. Our study targeted local and migrant households living in close proximity to the mining facilities in the community.

## Inclusion criteria

The following were taken into consideration during sampling:

- Age of adolescents: only households with adolescents aged 15–19 years were included in the study.
- Status of household: households headed by parent, siblings, grandparent, relatives and child-headed households were all eligible to participate.
- Distance from an active mine: only households within 20 km radius of an active mine were considered. The reason for this is because it has been speculated that living in close proximity to mine workers increases the risk of sexual risk behaviour among adolescents.
- Families and adolescents must have lived in the community for more than six (6) months, this is to ensure that participants are familiar with the environment.

## Population and sampling

The households of interest were those families with adolescents aged 15 to 19 years, including child-/adolescent- headed households. Purposive sampling method was used to identify suitable households for in-depth interviews. This was done in conjunction with community health workers who helped to identify at-risk households where adolescents have been known for risk behaviour. In addition, the participating households were selected based on their structural characteristics and closeness of household to the mines. Parents and adolescents in the selected house were eligible for sampling, the reason for this was to understand the family dynamics from different perspectives. Therefore, consenting parents/caregivers and their adolescents who met our selection criteria were interviewed.

## Data collection

An interview-guide which was based on the aim of the study was used to direct question during the interview. The main question asked was “Please can you tell me about your family”? Further questions were asked as a guide to further explore the family environment and its influence on adolescent sexual behaviour. This interview-guide was only a guide to keep the participants focus the aim of the study; in order words, the participants were allowed to express themselves. Data collection took place at the homes of selected families and each interview lasted for 45–60 min. The purpose of the study was explained to the participants before seeking their consent to participate in the study. Those who gave their consent were taken to a private and comfortable part of their house for the interview. In order to build trust among adolescents and encourage them to speak freely, we conducted the interviews in a sequence, the parents were interviewed first and their children were interviewed later. Participants were engaged in a one-on-one interview with the aim of uncovering their everyday activities as a family, this process continued until there was no new information coming from the participants and it was at this point that we stopped interviewing participants, and at this time, a total of 23 interviews had been conducted. Among the interviewees were 10 parents and 13 adolescents. The adult participants in this study were mainly females who had little or no formal education and were unemployed (Table I).

**Table I.** Demographic profile of participants

	Adolescent		Parents	
Gender	Male	3	Male	1
	Female	10	Female	9
Age	15–19	13	<50	6
			>50	4
Level of education	Student	8	<Grade 12	8
	Not attending	3	Grade 12 and above	2
	Completed grade 12	2		
Employment status	Employed	1	Employed	1
	Unemployed	3	Unemployed	7
	Self employed	-	Self employed	2
Relationship status	Single	13	Single	6
	Cohabiting	-	Cohabiting	2
	Married	-	Married	2
Religion	All Christians		All Christians	

Married men in this community were reluctant to participate in our study, most of whom referred the research team to their wives for interviews. The reason for this behaviour was not apparent but could be due to the sensitive nature of the study which sought to explore adolescents’ risk behaviour in the context of their family environment. It is possible that these men may feel judged since culturally, it is their responsibility to provide for and care for their families.

During the interview, we observed that some members of the family felt embarrassed about the question that they had to answer, while it was obvious that some tried to cover up and not tell the truth about what happens in their family, this was common among parents. On the other hand, adolescents were open and talked freely about the dynamics within their family, in some cases giving a completely different account of what their parents had said. In discussing our findings, the quotes from participants were presented to support our argument while literature served as control. The alphabets “P” and “A” were used to differentiate between the comments made by parents (P) and those made by adolescents (A) respectively.

## Data analysis

Using thematic analysis as described by Green and Thorogood (2009), we reviewed the data set thoroughly by listening to the recordings multiple times and also going through the field notes in other to make sense of the message. This was followed by transcribing the audio recordings beginning with the most interesting interviews. Interesting features in the transcripts and field notes were identified and coded systematically and the codes were later organized. The identified codes were grouped and from these groups we identified themes. The themes were reviewed against available data, those that did not have sufficient database to support them were collapsed and excluded. In line with the above steps, the following themes were generated, “Household poverty”, “Family conflicts”, “Lack of discipline”, “Parent-child closeness”, “Generational gap between adolescents and their parents” and “Lack of sex education”. Each theme was described to capture its true essence, and finally, the themes were organized to present a narrative that represents the data across themes.

## Measures of trustworthiness

In order to ensure the trustworthiness of our data, we applied the constructs of credibility, dependability, transferability and confirmability as proposed by Lincoln and Guba (1985).

We ensured credibility by identified participants who met the eligibility requirements, they were interviewed in a secure and comfortable place in their homes away from ear shots of people so as to create an environment conducive for disclosure. Furthermore, participants were engaged for a prolonged time. During this time, FCA observed the participants as they talked and made additional notes and further clarified questions to find out more about what is being said. HAA and NON reviewed the process of data collection, and where necessary, amends were made to ensure credible outcomes. Although it is difficult to make a general statement within the context of a qualitative research, the authors ensured transferability by applying proper and in-depth description of the context within which the research was approached. This is meant to provide a guide for a comparison of our findings in a different context or situations.

The authors ensured dependability by allowing cross-checking of codes by colleagues who were not part of the study to review the entire process that was applied during the study and matching these processes with the findings or outcome. Confirmability in this study was ensured by proper documentation of the procedures for checking and rechecking of data, and the attestation of the results by FCA, HAA and AKT, and the results were also compared with findings by other authors.

## Ethical consideration

The permission to conduct this study was issued by the Health, Safety and Research Ethics Committee of the University of Venda with project number SHS/16/PH/06/2305. The authors also secured approval from community leaders and ward counsellors in the index community.

Our participants included adults and adolescents, therefore, parents or guardian gave written consent on behalf of their adolescents before they were allowed to participate in the study. However, oral consent was given by adolescents before the interview. The purpose of the study was explained to the participants and their parent/parents and they were informed of their right to withdraw from the study should they feel uncomfortable with the process. For adolescents who were heads of household, the authors got consent from ward counsellors before the adolescents were interviewed. The participants were assured that the information provided by them and all forms of documentation received from them will be secured from the public domain. In addition, the participants were not asked to provide any form of identification by way of name and address.

## Findings and discussion

The families in the index community are faced with numerous challenges, some of these challenges were expressed during our interview. At the end of data analysis, the following themes emerged, “Household poverty”, “Family conflicts”, “Lack of discipline”, “Parent-child closeness”, “Generational gap between adolescents and their parents” and “Lack of sex education”. These themes relate to the opportunities and sometimes the missed opportunities within the family to address sexual risk behaviour among adolescents. For instance, issues relating to household poverty were seen to contribute immensely to sexual risk behaviour and the reason for this may not be far-fetched because where adolescents are not well taken care of financially, they may be lured to take a sexual risk in other to achieve their desired level of comfort. “Family conflict” as described by participants creates a negative energy within the home that would often lead to neglect of parental responsibilities which in turn may give rise to “Lack of discipline” among adolescents. Furthermore, family conflicts may also diminish “Parent-child closeness”—which is another theme generated from our study.

Generational gap between adolescents and their parents—this theme captured the frustrations expressed by participants pertaining to such issues as the age difference as well as the cultural changes between older parents (especially the grandmothers) and their adolescents. This generational gap creates a barrier to effective communication that limits the opportunity to provide sex education to adolescents.

## Poverty

Although poverty and inequality were wide-spread in the study community, its impact was worse felt among adolescents who lived by themselves and those who lived in households where the parents or caregivers were unemployed. This finding is well documented in literature where it has been argued that young people in resource-scarce communities face a great deal of challenges (UNFPA, 2012). As shown in the present study, the challenges faced by vulnerable adolescents were multi-faceted. These include poverty, loss of parents to the HIV and AIDS pandemic, parental separation, absent fathers and absent mothers, lack of social and emotional support and high levels of school dropout. One of the issues that resonated among the participants was the lack of financial support, even when such support was available, it was often not sufficient. Some of these young people work to be able to support themselves, and in some cases to support their families. One adolescent had this to say:

I don’t go to school, I stopped attending when my mother died. I do casual jobs to support myself and my grandmother but I also get support from my grandmother sometimes when she gets her grant money (A006, 16years)

Parental support has been judged to have the greatest influence on adolescent behaviour (Carlson, 2012). The amount of financial resources available to the adolescent can influence their health outcomes on many fronts, some of which include nutritional status, place of domicile and whether they engage in deviant behaviour or not (Amoateng, Kalule-Sabiti, & Arkaah, 2014). During our interview, participants reported that some adolescents were in dare need of financial, social and emotional support. This is evident in the following comments:

My adolescent granddaughter has no one to support her. Her mother is always sick and her father does not care. She is suffering, she does not get well taken care of like other children. I don’t want her to start misbehaving like other girls in this community (P007)

I don’t have support from anyone since my mother died, I don’t get any support from my elder brother with whom I live, that makes me to have many boyfriends so I can support myself (A008, 18years)

Child-headed households (CHH) expressed even deeper concerns regarding the state of their wellbeing, for instance, there were complaints of insufficient funds, lack of support with academic work, experience of deep emotions like sadness and anger. This assertion is corroborated by the work of Sumbulu (2014) where it was reported that the average monthly income available to CHH is around R1, 221.00 (75.33 Euro). According to this report, the household income of CHH could range from no income to about R 3,000.00 (185.10 Euro) per month. In line with this literature, we found that CHHs live in poverty and may also lack social support. The following comment was made by one adolescent participant:

We live on the rent of our shop which is about R800 (49.36 Euro) a month, and sometimes I do casual jobs. It is from this money that I take care of myself and my little sister”. My aunt used to support us but now she has relocated. (A011, 17years)

Unemployment, particularly among black communities in South Africa is a major contributing factor to household poverty (De Witte, Rothmann, & Jackson, 2012). Some of these households are plunged deeper into the trenches of poverty because they are graciously taking the responsibility of accommodating and taking care of their unemployed relatives (Klasen & Woodlard, 2009). This literature is corroborated by our findings which showed that in some instances, household poverty results from accommodating, feeding and caring for extended family members who have migrated to the index community in search of jobs that are either non-existent or not within the reach of applicants due to their low level of education, and the continued stay of these unemployed relatives impacts on the merger income of the breadwinner and by extension affects the financial support available to adolescents in the household.

The problem of unemployment is lopsided in favour of men (STATS SA, 2019). This was evident in the present study, where unemployment was common among female participants. Due to the high levels of unemployment in the index community, some parents, particularly the fathers have migrated to other locations in search of jobs, leaving behind their wives or adolescent children to care for themselves. This finding is corroborated by Ullah (2017) who argues that economic migration of breadwinners often results in reversal of gender roles, single parenthood and poverty. In the same light, De Witte et al. (2012) argue that unemployment results in negative affective experiences which include but not limited to worthlessness, conflicts, boredom and loneliness. The mentioned consequences of unemployment may inhibit youth empowerment and create a void which may be filled with self-destructive behaviours like risky sexual practices, drug abuse and violence (McNamara, 2003). Some young people may engage in unprotected sex in an attempt to survive, though they may be aware of the high-risk behaviour of their partners, but they do it anyways because of the financial gain they will receive from such a person (Graham, 2016). This assertion was supported by our findings where teenage girls reported having multiple sexual relationships in other to raise enough money to care for themselves. As documented in the literature, economically disadvantaged youths are more likely to engage in deviant sexual practices such as having sex in exchange for money or gifts and engaging in multiple sexual partnership. In our study, some female adolescents admit that the financial constraints in their families have compelled them to engage in risky sexual behaviour such as having multiple sexual partners.

Furthermore, our study revealed that even among those who were employed, financial constraint was a common predicament. Interviewed adults and adolescent members of households agreed that they are often unable to meet their financial obligations to their children and siblings. The worst affected were the child-headed households and homes where the grandmother is the breadwinner. This situation gives credence to the argument that poverty has a grave influence on parenting in South Africa, according to Kaiser, Li, Pollmann-Schult, and Song (2017), it limits the intent of parents to raise their children within an environment that excludes harshness and emotional abandonment. In view of this predicament, the South African government has moved to support disadvantaged families with fund transfers in the form of old-age pension and child support grant (Cluver et al., 2013). Arguably, it has been speculated that the provision of these grants, especially the child support grant, has reduced the likelihood of adolescent girls engaging in transactional sex and sexual relationships with older men (Cluver et al., 2013), but interestingly, it appears that this is not the case (Klasen & Woodlard, 2009).

I struggle to meet my responsibility of feeding my grandchildren because my grant money is not enough, even when I included the children’s grant, it is still not enough. My son (47 years old) who is not working is also living with me and the children. So it is difficult to take care of the needs of my grandchildren especially those that are grown (P007)

## Family unity

We found compelling evidence to suggest that couple conflict was a common phenomenon in the index community, and this was particularly so among single mothers. This is not to say that married couples did not experience conflicts in this community but the phenomenon was deeply expressed by single and cohabiting couples and the adolescents who live with them. These are some of the comments by participants:

My older sister (the one I live with) and her boyfriend are always fighting. He drinks a lot and he is always fighting with her, this makes me unhappy. Sometimes I wish I can get away from the house but I don’t have a choice (A007, 15years).

This finding is important not only because it corroborates previous research findings (Reynolds, Houlston, Coleman, & Harold, 2014) but because of the negative impact couple conflicts have on adolescents’ behaviour (Branje, 2018). For instance, it has been documented that couple conflicts diminish parents’ ability to effectively care for their children, and if such conflicts remain unresolved, it has the potential to strain parent–child relationship (Branje, 2018). A parent in conflict with a spouse is likely to be aggressive and hostile to a child or become lax or uninterested in the activities of the child and both of these spectra of parenting pattern supports untoward health outcomes for the child (Reynolds et al., 2014). This assertion is supported by the following quote:

In this community, it is difficult to train children. Some parents are not behaving well … .some of them (adolescent), their parents have failed them, some parents are drinking and fighting in front of their children and this may affect their upbringing. (P008)

An interesting but not surprising finding in our study was that whilst parents denied any form of conflict with their partners, their adolescents revealed that their parents or their mother and her partner/s were constantly fighting. Our findings illuminate efforts by parents to cover up uncomfortable and embarrassing situations in their households that may negatively influence adolescents’ behaviour, this is significant because parents are supposed to be role models to their children. Through observation or by direct teaching of cultural norms and traditions, children learn a lot from their parents and other adult members of their family. Therefore, it suffices to say that children are often misguided when the family is unstable or the parents are absent, emotionally distant or the parents themselves are delinquent. This gives credence to the argument that inadequate parental monitoring increases the likelihood of an adolescent engaging in early sexual risk behaviour (Coley, Votruba-Drzal, & Schindler, 2009).

## Discipline

The circumstances surrounding families in this community make it difficult for parent/s to meet their obligations to their children. Those who are gainfully employed in the mining industry are torn between keeping their social responsibility to their families and keeping up with their job demands. Some of these mine workers live in hostels in the mines which are designed to accommodate only staff members but not their families. Therefore, most of the time they are away at work, leaving the responsibility of raising the children to their spouses or relatives. On the other hand, those who are unemployed grapple with finding some means to provide for their families. This situation makes it difficult for such parents to monitor and exercise control over the activities of the children, especially the adolescents.

Furthermore, we found that parents were indiscreet with their alcohol use and sexual risk behaviour, therefore exposing their children to risk factors for untoward behavioural outcomes, these practices limit parent’s opportunities to control and discipline erring adolescents. In addition, grandmothers who participated in our study were frail, some even suffered from medical conditions that restricted their ability to effectively monitor and control their adolescent children.

The children in this community lack good home training because their parents are badly behaved. Badly behaved parents breed badly behaved children (P002)

It is only reasonable to expect that children who live with parents who support them emotionally would generally do better than those who do not have such support (Holborn & Eddy, 2011). Furthermore, families where the father is either dead or absent have been shown to struggle with maintaining discipline and monitoring of adolescents behaviour (Kimani & Kombo, 2010). These families are also more likely to struggle with providing food, clothing and paying of school fees (Kimani & Kombo, 2010). Another striking challenge as far as household discipline goes is the difficulty experienced by the head of a CHH in maintaining law and order in the home, this may be the case when the age difference between the head of household and the sibling is marginal. One head of the household said it is difficult to get my siblings to obey me. She would have to plead and sometimes offer financial incentives before she can get them to assist with chores at home. This kind of disputes are common in CHH but not peculiar to it, this is corroborated by Mthethwa (2009). Furthermore, the heads of households are at liberty to do whatever they like, after all, they set the rules at home and no one can tell them what to do. Therefore, the behavioural outcome in the family may depend on the conduct of the head of household. Conversely, it is important to note that the conduct of children in CHH is not always destructive (Sumbulu, 2014). It is often expected that in the absence of parents or an adult, children in this kind of settings would become wayward and destructive but this is not always the case; for example, some of them have lost both their parents to the HIV and AIDS pandemic (Germann, 2005) and are aware of the consequences of engaging in risky behaviour. Such adolescents are careful and vigilant. Our study illustrates this with the comments of one participant:

I am in charge of the house, I do what I like. I have friends, some have good behaviour while some drink, smoke and go to taverns all the time, but they have not been able to change me to behave badly (A011, 17years)

Parents in our study complained about bad behaviour among children living in this community, for some, the complaint was that the children were disrespectful, while for others it was that the children engaged in early sexual activities, drinking alcohol, smoking marijuana and using other harmful substances. The ability of some of the households to set rules and maintain discipline is diminished due to several reasons. For example, some households are headed by elderly grandmothers who are not able to monitor or control their grandchildren because of their own medical conditions, while in some households, there are no adults to monitor adolescents and these young people automatically become decision-makers in their households. In some households, there is a single mother who is limited in her ability to maintain law and order in the home.

There is evidence to support authoritative parenting style as the most protective with regards to adolescent risk behaviour compared to authoritarian and permissive parenting styles (Becona et al., 2012; Domenech Rodriłguez et al., 2009). The present study found that this is not always the case, some participants expressed support for authoritarian parenting style where the parent is seen as being overtly strict. For instance, one participant expressed that her parents are authoritarian and she believes she has turned out to be better because of that. This is what an adolescent had to say:

I can describe them as strict parents, they even punish me when I disobey, I believe my behaviour is good because of it. (A013 18years)

Broadly speaking, the assertion that authoritative parenting supports healthy adolescent behaviour may have been made because this type of parenting style creates an environment that encourages communication. However, Huebner and Howell (2003) found no relationship between authoritative parenting style and the level of sexual risk-taking, but parental permissiveness has been shown to enhance opportunities for substance use and sexual risk behaviour (Donenberg, Emerson, Bryant, & King, 2006). On the one hand, parents who set rules and monitor them are likely to reduce early sexual initiation, and unsafe sexual behaviour (Coley et al., 2009; O’Donnell et al., 2008). On the other hand, adolescents who are not properly monitored are at increased risk of delinquent behaviour and unsafe sexual practices (Caldwell, Beutler, Ross, & Silver, 2006). Furthermore, when parents are harsh and inconsistent with their punishment, they reinforce bad behaviour (Laghi, Baiocco, D’Alessio, & Gurrieri, 2009; Tafà & Baiocco, 2009).

## Parent—adolescent closeness

In our study, we found that parents and their adolescents claimed that they enjoyed a close relationship with each other. Although several adolescents agreed that they were close to their parents but they also claimed that being close to their parents did not influence their behaviour. On the other hand, some parents claimed that they had a good relationship with their children but these claims were refuted by their children. It appeared that the bonding involved in these relationships among our participants was not strong because as much as they claimed that they were close to each other, adolescents were quick to say that being close to their parents had no influence on their behaviour. According to Oswalt (2015), parent–child closeness is an important factor in fostering healthy adolescent development. However, it has been shown that the closeness between parents and their children begins to wane as the children begin to attain adulthood (Oswalt, 2015). The effect of this has been argued back and forth; some say that even as the child grows older, the relationship with parents remains an import source of social and emotional support (Collins & Steinberg, 2007) while others argue that the protective effect of this relationship on adolescent behaviour may also diminish as the child grows older (O’Donnell et al., 2008). However, some authors posit that the effectiveness of the parent–child relationship on adolescent risk behaviour depends on the quality of such relationships (Newman, Harrison, Dashiff, & Davies, 2008). Our findings support claims that the effect of parent–child relationship on positive adolescent behaviour may dwindle as adolescents get older. This is because some adolescents in our study who reported having a close relationship with their parents also reported engaging in risky sexual behaviour, such as multiple sexual partnership, alcohol binging and even teenage pregnancy. However, this was a common occurrence among older adolescents.

My closeness to my mother does not change my behaviour, my behaviour depends on me. (A003, 18years)

On the other hand, some adolescents reported that they did not enjoy a good relationship with their family. This was common among adolescents in a sibling-headed household. This is what one adolescent had to say:

I don’t enjoy a good relationship with my elder brother, we are not close and he does not support me that’s why I prefer to live with my aunt. (A008, 18years)

## Generational gap

In the present study, both groups of participants (parents and adolescents) identified age and cultural gaps as hindrances to effective communication between parents and their children. This was particularly common among households headed by grandmothers. Some participants expressed the view that the cultural values of the society are being eroded by modernization and the older generation is overwhelmed by the behaviour of young people. Similarly, some young people do not comprehend why older people do not share their sentiments regarding certain behavioural decisions that they make. Adolescents in the present study expressed discomfort sharing their personal problems with their parents, some say they are afraid of what their parents might say, while some worry that their parents would forbid them from dating. This is what some adolescents had to say:

As an African child, we don’t often discuss with our parents certain topics but I do discuss my personal issues with my friends and not my grandmother. I don’t discuss my sexual life with her because she might not understand”. I don’t discuss such things with my grandmother because of the age difference between us (A009, 18years)

Similar sentiments where expressed among grandmothers, a particular grandmother said the following:

I don’t encourage my grandson to discuss his personal things with me, I don’t want to encourage him to start thinking about those things (sex). Besides, he is still too young for those kind of things (P004)

It has been suggested that adolescence is a stage for self-discovery, a time when young people begin to form bonds with peers (WHO, 2014a) and gradually shifting their source of primary social support away from their parents to their peers (Oswalt, 2015). Parents who remain distant from their children may create an opportunity for adolescents to bond strongly with their peers who may influence them negatively (O’Donnell et al., 2008). It has been documented that adolescents are more likely to disclose their personal and intimate activities to their parents only when they believe that their parents will give them the attention they desire and when such intended activities will not be disapproved by their parents (Yau, Tasopoulos-Chan, & Smetana, 2009). Adolescents are also more likely to disclose when they perceived that there is warmth, support and understanding from members of their family (Somers & Vollmar, 2006).

## Sex education

Adolescents who were interviewed during this study complained that there was little or no sex education from their parents or caregivers. Some say the only time their parents talk about HIV or pregnancy to them is when they were being scolded for bad behaviour. However, it is important to note that this complaint was common among adolescents in grandmother-headed household. This is what one participant had to say:

Sex education is not enough at my home, my grandmother is so strict but she only advise me about sex when I offend her. (A001, 15years)

The environment within which young people live today is different, adolescents feel uncomfortable discussing sex with their parents, and parents on the other hand are either not willing or uncomfortable to initiate such discussions (Nambambi & Mufune, 2011). Although parents agree that children need to be educated about sex, some believe that adolescents have already been exposed to sex through different platforms ranging from television viewing, magazines, billboards and social media. They believe adolescents already know more than they are supposed to know (Dyson, 2010). Some parents are of the view that the school system would be in a better position to give more evidence-based teaching about sex (Dyson, 2010). This is supported by arguments that parents might be limited in depth and scope because some parents do not have the requisite knowledge on contemporary issue about sex (Lukolo & van Dyk, 2015). However, Shtarkshall, Santelli, and Hirsch (2007) argue that promoting healthy adolescent sexuality is not to be strictly a responsibility for parents or school teachers but rather a collective responsibility of both. During our interview, it was evident that parents were really uncomfortable talking about sex, especially among older parents (those above 45 years of age), as some of them could not respond to our questions directly. This is what a parent had to say:

I do not talk about sex that much with my child. I believe the teacher is doing that at school (P002)

However, it has been shown that frequent communication about sex with parents significantly reduced the influence of sexually active friends and experienced peer pressure on adolescents’ intention to have sex (van de Bongardt, de Graaf, Reitz, & Dekovic, 2014). Furthermore, the negative influence that peers have on adolescent sexual behaviour may be diminished with effective parent-child communication about sex (van de Bongardt et al., 2014). This is premised on the fact that parents will provide accurate information about sex than peers. In addition, children who spend more time talking to their parents about sex and the use of condoms would most likely spend less time with their peers, thereby reducing opportunity for peer influence (Ryan, Roman, & Okwany, 2015).

## Conclusion

Although studies have shown that adolescents’ behaviour can be influenced by both familial and extra-familial factors, it is believed that the family system has more influence on the adolescent. Adolescents who live within a secure family system where the parents are united, morally and financially stable are more likely to show good behaviour in the face of changing environmental factors. Our study identified several factors within the family that might have a significant influence on adolescent’s sexual risk behaviour. We revealed that families living in these communities are faced with numerous challenges, ranging from couple conflicts, poverty, absent parent/s and a volatile physical environment which remains a source of negative influence on adolescents. In addition, we also reported poor parent-adolescent bonding, poor communication and delinquency among some parents. These challenges weaken the support system within the family that otherwise would provide needed guidance to the adolescents. It might be on this premise that the adolescents are lured into early and risky sexual behaviour.

## Implication

Our findings suggest that the factors associated with sexual risk behaviour are multifaceted and therefore, a multipronged approach is needed to improve the health outcomes of adolescents in migration-affected communities. Therefore, it is imperative that community-specific studies like the index study are harnessed in order to develop evidence-based public health programmes to support adolescents and help them make informed choices regarding their sexual health.
